# Prevalence of Referred Pain with Pulpal Origin in the Head, Face and Neck Region

**Published:** 2008-04-02

**Authors:** Siamak Mardani, Mohammad Jafar Eghbal, Maryam Baharvand

**Affiliations:** 1 *General Practitioner, Tehran, Iran*; 2 *Department of Endodontics, Iranian Center for Endodontic Research, Dental Research Center, Dental School, Shahid Beheshti University of Medical Sciences, Tehran, Iran*; 3 *Department of Oral Medicine, Dental School, Shahid Beheshti University of Medical Sciences, Tehran, Iran*

**Keywords:** Dental Pulp, Endodontic, Referral Pain

## Abstract

**INTRODUCTION:** This study was designed to evaluate the prevalence of referred pain with pulpal source in the head, face and neck region among patients referred to Dental School of Shahid Beheshti University MC, Tehran, Iran in 2004.

**MATERIALS AND METHODS:** In this cross-sectional study, 100 patients (55 males and 45 females) referred to oral medicine department of Shahid Beheshti Dental School evaluated via clinical and radiographic examination to seek their pain sources and sites. Inclusion criteria were report of pain and a dental clinician accomplished detection of pain origin. Exclusion criteria were non-odontogenic painful diseases, advanced periodontal disease, and substantial carious lesions. Visual analogue scale (VAS) was used to score pain intensity; meanwhile the patients were asked to mark the painful sites on an illustrated head and neck mannequin.

**RESULTS:** Sixty-five percent of patients reported pain in sites which diagnostically differed from the pain source. According to statistical analysis, duration (P<0.01), spontaneity (P<0.001) and quality (P<0.01) of pain influenced its referral nature, while sex and age of patients, kind of stimulus, throbbing and intensity of pain had no considerable effect on pain referral (P>0.05).

**CONCLUSION:** The prevalence of referred pain with pulpal origin in the head, face and neck region is moderately high which requires precise diagnosis by dental practitioners. Some hallmarks of irreversible pulpitis (*e.g*. spontaneous and persistent pain after elimination of stimulus) are related to pain referral.

## INTRODUCTION

Referred pain is a common and confusing problem every dental practitioner may encounter (1). This is a kind of pain perceived in a part of body, which is far from the source of pain. Usually the pain originated in a visceral organ could be referred to a superficial anatomic region such as cardiac pain, which radiates to the shoulder, arm, mandible and face (2-3). Referred pain may also be detected in the face and teeth e.g. a toothache may be referred to non dental anatomic structures and vise versa pain from other regions may be perceived in teeth (4-6). Many theories have been proposed to explain referred pain such as “convergence theory” and “expansion of receptive fields” (7).

Obviously, a successful dental treatment requires detection of the source of pain. If the origin of pain is not found it may lead to inappropriate dental care like extraction or root canal therapy. Meanwhile, pain originated from other anatomic sites like masticatory muscles and mucosa will not be relieved by extraction or endodontic treatment. These illogical therapies, unfortunately, are very common as attempts to decrease pain, but they are not only ineffective but also cause more complications for patients and legal affairs for dentists (8).

Falace *et al*. surveyed 400 patients attended at an emergency dental clinic in Kentucky with posterior tooth pain. The referred pain was reported to be 89.9%. The most common site for referred pain was neighboring teeth (80%), and the frequency of pain radiating to opposite dental arch was 24%. The results of Falace’s study revealed that the intensity of pain is significantly correlated to its referral nature while the duration and quality of pain have few effects on it (7).

**Table 1:** Pain duration related to pain referral status

Pain	Number	Mean of pain duration
Referred	65	26 days
Non-referred	35	62.2 days

Referred pain is frequently distinguished by local irritation and elective local anesthesia. In other words, whenever the pain referral, local irritation such as, heat and percussion do not increase pain, and local anesthesia in site of pain does not alleviate it as well (9).

However, referred pain may interfere with diagnosis as the basic step for treatment. They do not appear following a uniform pattern and new referral modalities may always be encountered. Meanwhile, inadequacy of researches regarding referred pain makes it necessary to design studies about similar subjects. The objective of this study was to determine the prevalence of referred pain with pulpal origin in the head, face, and neck region in patients referred to Dental School of Shahid Beheshti University MC, Tehran, Iran in 2004.

## MATERIALS AND METHODS

One hundred patients (55 males, 45 females), reporting pain in the head, face and neck region were studied. Subjects were recruited from patients referring to oral medicine department of Dental School of Shahid Beheshti University MC, Tehran, Iran, during May and June 2004. Inclusion criteria were: 1) Patient’s report of pain in head and neck region; and 2) Pulpal origin for pain confirmed by diagnostic aids.

Exclusion criteria were: 1) History or presence of non-odontogenic painful diseases in the region; 2) Existence of advanced periodontal disease, which may cause pain; and 3) Multiple carious lesions that make it difficult to consider a distinct pulpal origin for the pain.

A questionnaire filled including demographic information, medical history, and characteristics of pain resulted in diagnosis of source and site of pain. After interview and clinical examination, practitioner’s clinical verification and finally appropriate radiographs (mostly periapical) were taken to confirm the diagnosis and the interpretation was recorded on the questionnaire. Searching for pain source began using subjective reports of patients, clinical verification and radiographic interpretation.

All subjects underwent diagnostic procedures and subsequently a definite diagnosis was obtained for each case. After recognition of pain source and site, if they were the same, the pain considered as primary (or non-referred); otherwise, it was considered as a referred pain. Furthermore, to measure pain intensity, visual analogue scale (VAS), as a world wide accepted method, was applied. In addition, patients were asked to draw their site of pain on a mannequin. The mannequin was depiction of head and neck region from different views. Moreover, the chi- square test was used to detect the association of referred pain incidence with verbal description of its characteristics (*i.e*. spontaneous or provoked, dull or sharp, throbbing or not and longevity of provoked pain) and kind of stimulus (*e.g*. thermal or pressure). Also, two-sample t- test in two modes of equal and unequal variances was used to find out the association of pain referral with quantitative variables (*i.e*. pain intensity and duration). At the end, logistic regression was applied to test the degree of association of referral pain with age of subjects.

## RESULTS

Among 100 patients (55 males and 45 females), ranging from 14 to 66 years old, with pulpal pain 65% (n=65) had experienced pain referral to head, face and neck region. Mean of pain duration was 26 days for these subjects and there was a statistical relation between pain duration and its referral status (P<0.01) (Table 1). Among patients with referred pain, 7.7% had spontaneous pain, 20% had provoked pain and 72.3% had both of them, (Table 2). 83.3% reported lingering and 16.7% announced brief provoked pain; %76.9 had experienced dull while others had sharp pain. All of pain characteristics had significant association with pain referral status (P<0.01), while kind of stimulus like temperature (*i.e*. cold and warm irritation), mastication and pressure had no effect on prevalence of referred pain. Likewise throbbing pain showed no meaningful relation with its referral nature. Mean pain intensity according to VAS among those with referred and non-referred pain was 7.6 and 6.6, respectively. Statistically, intensity of pain didn’t have any association with its referral nature. Pain referral didn’t show any difference to occur in different age groups.

**Table 2:** Pain referral status related to pain qualities

PAIN	Referred		Non-Referred		Total	
Characteristics	No	%	No	%	No	%
Spontaneous	5	7.7	0	0	5	5
Provoked	13	20	20	57.1	33	33
Both of the above	47	72.3	15	42.9	62	62
Brief	10	16.7	19	54.3	29	30.5
Lingering	50	83.3	16	45.7	66	69.5
Sharp	15	23.1	17	48.6	32	32
Dull	50	76.9	18	51.4	68	68
Temperature provoked	10	16.7	7	20	17	17.9
Mastication &pressure provoked	7	11.7	6	17	13	13.7
Both of the above	43	71.6	22	62.9	65	68.4
Throbbing	37	56.9	19	54.3	56	56
Non-throbbing	28	43.1	16	45.7	44	44

## DISCUSSION

In this study the prevalence of referred pain was 65% in comparison with Falace’s study in which 89.8% of samples reported referred pain (7). The difference may be due to study design. We used radiographic imaging as a powerful diagnostic aid to detect pulpal pathosis unlike Falace’s study in which only subjective reports and clinical verification were used, so even mild cases of pulpal involvement (reversible pulpitis) which may less frequently cause referred pain were included in our study and this may lead to lower overall estimation of referred pain prevalence.

In addition, we found out a relationship between the duration of pain and its referral nature, *i.e*., in cases with referred pain the mean duration of pain was lower (26 days) than that of non- referred pain ones (62 days) because the latter is more tolerable and this was contrary to Falace’s study (7). The mean duration of 26 days for referred pain seems a little long. This may be due to dentophobia, economic problems or consumption of analgesics, which may cause delay to visit a dentist.

## CONCLUSION

Over one half of dental patients with pulpal involvement have referred pain to face and head region, which mandates exact diagnosis. The intensity of referred pain was higher, and the duration of it was shorter than that of non- referred pain.
